# Lipodystrophy, Diabetes and Normal Serum Insulin in PPARγ-Deficient Neonatal Mice

**DOI:** 10.1371/journal.pone.0160636

**Published:** 2016-08-09

**Authors:** Peter E. O’Donnell, Xiu Zhen Ye, Melissa A. DeChellis, Vannessa M. Davis, Sheng Zhong Duan, Richard M. Mortensen, David S. Milstone

**Affiliations:** 1 Vascular Research Division, Department of Pathology, Brigham & Women’s Hospital and Harvard Medical School, Boston, MA, United States of America; 2 Department of Physiology, University of Michigan Medical School, Ann Arbor, MI, United States of America; Virgen Macarena University Hospital, School of Medicine, University of Seville, SPAIN

## Abstract

Peroxisome proliferator activated receptor gamma (PPARγ) is a pleiotropic ligand activated transcription factor that acts in several tissues to regulate adipocyte differentiation, lipid metabolism, insulin sensitivity and glucose homeostasis. PPARγ also regulates cardiomyocyte homeostasis and by virtue of its obligate role in placental development is required for embryonic survival. To determine the postnatal functions of PPARγ in vivo we studied globally deficient neonatal mice produced by epiblast-restricted elimination of PPARγ. PPARγ-rescued placentas support development of PPARγ-deficient embryos that are viable and born in near normal numbers. However, PPARγ-deficient neonatal mice show severe lipodystrophy, lipemia, hepatic steatosis with focal hepatitis, relative insulin deficiency and diabetes beginning soon after birth and culminating in failure to thrive and neonatal lethality between 4 and 10 days of age. These abnormalities are not observed with selective PPARγ2 deficiency or with deficiency restricted to hepatocytes, skeletal muscle, adipocytes, cardiomyocytes, endothelium or pancreatic beta cells. These observations suggest important but previously unappreciated functions for PPARγ1 in the neonatal period either alone or in combination with PPARγ2 in lipid metabolism, glucose homeostasis and insulin sensitivity.

## Introduction

Peroxisome proliferator activated receptor gamma (PPARγ) was discovered as an important ligand-activated transcription factor and pleiotropic regulator of adipocyte differentiation and lipid metabolism [1]. PPARγ functions in insulin sensitivity and glucose homeostasis [2] also suggest a prominent role in the metabolic syndrome, or syndrome X, a frequently occurring constellation of pathophysiologic abnormalities including obesity, insulin resistance, and dyslipidemia associated with type 2 diabetes mellitus, hypertension and atherosclerosis [3, 4]. In addition to its important functions in adults, PPARγ also plays a crucial role during placental vascular development. Mice lacking PPARγ die at midgestation with abortive differentiation of the placental labyrinth and failure to form the primary maternal-fetal vascular exchange interface ([5, 6] and unpublished observations).

Our goal is to determine the postnatal roles of PPARγ by a loss-of-function experimental strategy. However, pharmacologic inhibitors have not been suitable due to lack of specificity and potency [7], and placental failure precludes studies of non-conditional loss-of-function. Therefore, in our initial approach we analyzed embryonic stem cell/blastocyst-derived mice that were chimeric for homozygous PPARγ deficiency [8]. These experiments confirmed a specific and obligate role for PPARγ in adipocyte differentiation and adipose tissue development *in vivo* [9] and helped define PPARγ’s role in cholesterol metabolism by macrophage [10]. Subsequently, we and others used Cre-*loxP* to investigate cell-type specific loss of PPARγ function in adults. These studies revealed that: cardiomyocyte PPARγ participates in cardiac hypertrophy [11]; adipocyte PPARγ is required for normal adiposity [12–14] and for insulin sensitivity in fat and liver, but not in muscle [12]; skeletal myocyte PPARγ is required for normal adiposity and for insulin sensitivity in liver, but not in fat or muscle [15, 16]; hepatic PPARγ is required to maintain whole body insulin sensitivity, particularly in older animals or in genetically diabetic backgrounds, and mediates hepatic steatosis [17, 18]; endothelial PPARγ is important in diet-induced hypertension [19] and lipid metabolism [20]; and mice with PPARγ-deficient pancreatic beta cells show normal glucose homeostasis and retain antidiabetic responses to rosiglitazone despite showing islet hyperplasia on a chow diet and blunted islet expansion on a high fat diet [21]. Isotype-related functions of PPARγ have also been determined. Global deficiency of PPARγ2, the predominate isoform expressed in adipocytes [22], with retention of PPARγ1 expression mimics adipocyte-specific deficiency of all PPARγ isoforms [23]. Isoform-specific deficiency of PPARγ1 has not been reported.

Taken together, these studies began to elucidate the tissue and lineage-restricted functions of PPARγ and its isoforms. However, the effects of generalized PPARγ deficiency in the postnatal period remained unknown. Determining such effects is important for understanding how tissue-restricted and isoform-specific functions are integrated, which effects predominate, and which effects are rate limiting in different physiologic and pathophysiologic settings. Therefore, to determine the effects of generalized PPARγ deficiency *in vivo*, we used the MORE-Cre (Mox2-Cre) allele [24] to conditionally delete PPARγ in epiblast-derived tissues while sparing placental trophoblast. This approach rescued PPARγ-deficient placental failure and produced live born mice with severe, generalized PPARγ deficiency. Studies with these animals confirmed PPARγ’s role in adipocyte differentiation, revealed new effects in glucose, insulin and lipid metabolism, extended the appreciation of PPARγ function to circadian rhythms of behavior and metabolism and documented the feasibility of analyzing PPARγ function in adults by genetic loss-of-function [25–27]. Subsequent experiments substituting Sox2^cre^ for MORE^Cre^ to achieve more complete PPARγ deletion in epiblast confirmed rescue of midgestation embryonic lethality while administering PPARγ agonists produced fetal growth restriction with altered trophoblast and microvascular morphogenesis [28]. Angiogenesis-related gene expression was also altered in the latter stages of pregnancy. We now report that PPARγ-deficient neonatal mice born from MORE^Cre^-rescued gestations show near complete absence of adipose tissue at birth, severe alterations in lipid and glucose metabolism with functional insulin deficiency and diabetes and failure to thrive usually resulting in death by the second week of life. These findings suggest important but previously unappreciated consequences of PPARγ1 deficiency in the neonatal period either alone or in combination with PPARγ2 in lipid metabolism, glucose homeostasis and insulin sensitivity.

## Results

### Epiblast-restricted PPARγ deficiency rescues placental failure and reveals neonatal lethality

Breeding PPARγ^**+/-**^ x PPARγ^**+/-**^ mice did not yield any PPARγ-/- neonates among more than 500 live births (Table 1A) [5, 6]). In contrast, breeding PPARγ^F/F^ females to PPARγ^+/-^:MORE^+/Cre^ males yielded substantial numbers of live PPARγ^-/F^:MORE^+/Cre^ newborns (Table 1B). 95% of the floxed PPARγ allele in adults that survive from these matings was recombined to the null allele [25]. More extensive recombination is expected in mice that die during the neonatal period such that these adult results likely underestimate the amount of recombination in the neonates studied here that succumb. In addition, PPARγ mRNA was present but at very low levels in the small amounts of periuterine adipose tissue found in adult female mutant mice [25] suggesting that any such cells in these mosaic adults or neonates likely represent escape from recombination.” Therefore, conditional epiblast-restricted recombination directed by the MORE^Cre^ allele efficiently rescued embryonic lethality as previously reported [25] and similar to the results reported using Sox2^cre^ [28]. However, both parental genotype matings produce fewer than expected neonates bearing placental tissues with heterozygous deficiency for PPARγ (PPARγ^-+-^ in Table 1A or PPARγ^-/F^ in Table 1B) [29, 30].

**Table 1:** Epiblast-restricted recombination rescues PPARγ-deficient embryonic lethality.

**A**				
**PPARγ**^**+/-**^ **x PPARγ**^**+/-**^				
**PPARγ**	**O**	**E1**	**E2**	
**+/+**	**194** **38%**	**127** **25%**	**168** **33%**	
**+/-**	**314** **62%**	**254** **50%**	**340** **67%**	
**-/-**	**0** **0%**	**127** **25%**	**-**	
**B**				
**PPARγ**^**F/F**^ **x PPARγ**^**+/-**^**:MORE**^**+/Cre**^				
	**MORE**			
	**+/+**		**+/Cre**	
**PPARγ**	**O**	**E**	**O**	**E**
**+/F**	**127** **27%**	**117** **25%**	**141** **30%**	**117** **25%**
**-/F**	**96** **21%**	**117** **25%**	**104** **22%**	**117** **25%**

For each genotype the tables show the number and percent of live born neonates observed (O) and expected assuming Mendelian inheritance (E). (A) PPARγ^**+/-**^ female x PPARγ^**+/-**^ male. (B) PPARγ^F/F^ female x PPARγ^**+/-**^:MORE^+/Cre^ male. E1, expected assuming survival of all gestations until genotype determination. E2, expected assuming PPARγ^**-/-**^ embryonic lethality. Global PPARγ deficiency is uniformly fatal prior to birth. In contrast, substantial numbers of PPARγ^-/F:^MORE^+/Cre^ neonates are born with epiblast-restricted PPARγ deficiency. However, fewer neonates than expected are observed from PPARγ haploinsufficient placentas in the absence of MORE^Cre^ (PPARγ^**+/-**^ and PPARγ^-/F^ in A and B, respectively). A. p < 0.0001 *vs*. E1; p = 0.014 *vs*. E2. B. p = 0.012, 0.021 and 0.044 observed *vs*. expected for all genotypes, for mice inheriting Cre and for mice not inheriting Cre, respectively [29, 30].

**Fig 1:**
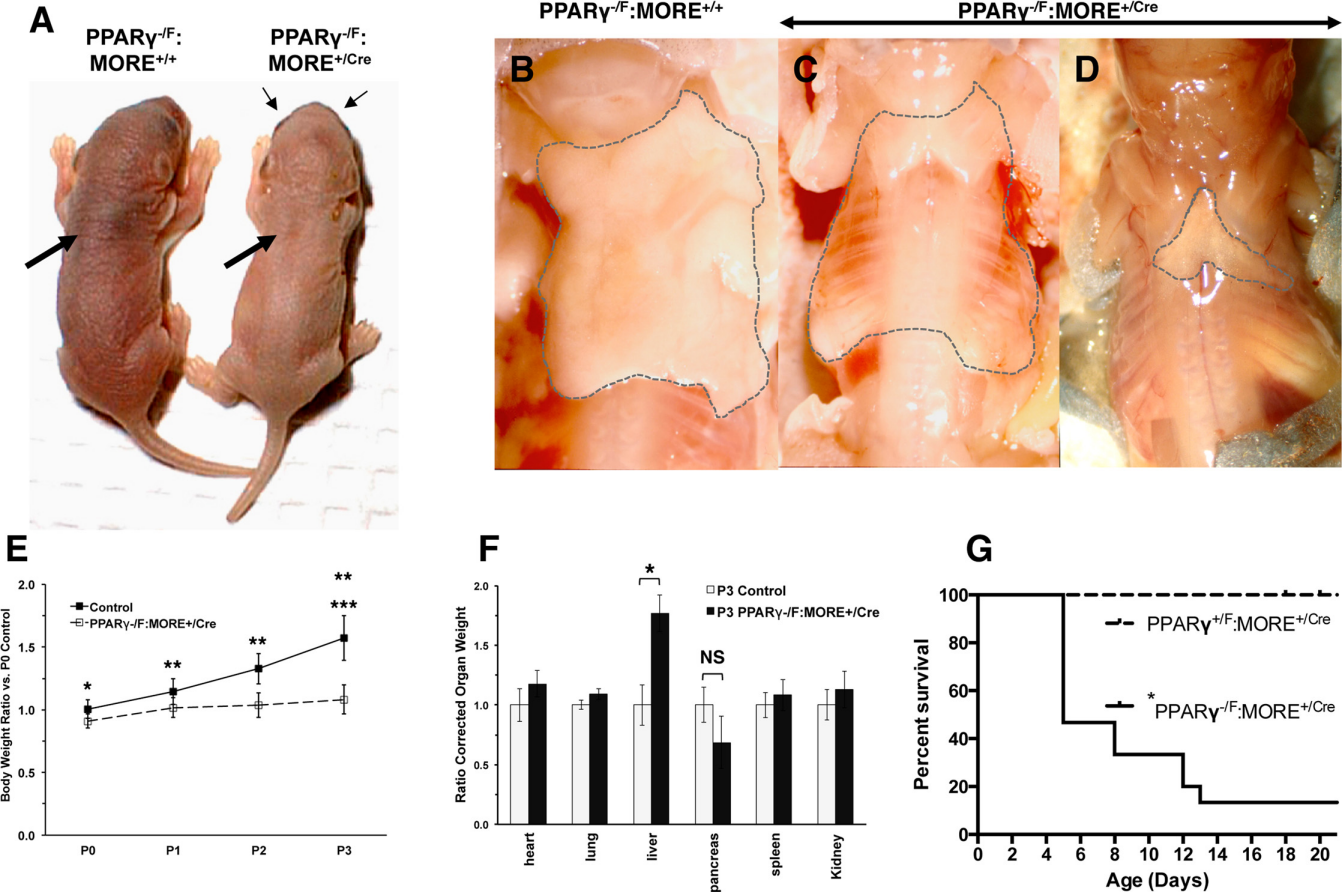
Failure to thrive and neonatal lethality in PPARγ^-/F^:MORE^+/Cre^ mice. (A) PPARγ^-/F^:MORE^+/Cre^ mice (right) show dermal hypopigmentation and an interscapular concavity (thick arrow, right panel) corresponding to the position of the interscapular brown fat pad in normal mice (thick arrow, left panel). Thin arrows designate retinal pigment indicating the paleness of this PPARγ^-/F^:MORE^+/Cre^ neonate is not due to albinism. (B-D) Interscapular adipose tissue was virtually absent in PPARγ^-/F^:MORE^+/Cre^ P3 neonates. The interscapular region contains BAT in control (dashed outline, B), but not in mutant (dashed outline, C) P3 neonates. Occasional mutants retained small amounts of interscapular tissue consisting primarily of white adipose tissue (WAT)-like tissue (dashed outline, D). (E) Body weight of neonatal mice from PPARγ^F/F^ x PPARγ^**+/-**^:MORE^+/Cre^ matings. PPARγ^-/F^:MORE^+/Cre^ mice are slightly smaller than littermates at birth (* p < 0.002), subsequently fail to gain weight in spite of apparently normal suckling and the appearance of a milk spot and are markedly smaller than littermates at P3 (*** p = 0.00001). In contrast, littermates gain weight daily (** p < 0.0003 vs. previous day). (F) Organ weights showing selective hepatomegaly in P3 PPARγ^-/F^:MORE^+/Cre^ mice (* p < 0.05; NS, p > 0.05). (G) Neonatal lethality of PPARγ^-/F^:MORE^+/Cre^ mice. The majority of PPARγ^-/F^:MORE^+/Cre^ mice were euthanized at humane endpoints or found dead between P5 and P13 (* p < 0.0001). In E and F “Control” indicates combined results for PPARγ^+/F^:MORE^+/+^, PPARγ^-/F^:MORE^+/+^ and PPARγ^+/F^:MORE^+/Cre^ neonates.

PPARγ^-/F^:MORE^+/Cre^ neonates were identifiable by gross inspection at birth displaying dermal hypopigmentation and a prominent interscapular concavity (Fig 1A). The latter reflected absence, or near absence of brown adipose tissue (BAT) (Fig 1B–1D). Occasionally, a small amount of residual tissue (as little as 5 mg could be appreciated visually) was present (Fig 1D). In addition, PPARγ^-/F^:MORE^+/Cre^ newborns were 9% smaller than control littermates at birth (p = 0.002, Fig 1E) and, despite successful nursing as judged by the presence of gastric milk, failed to gain weight postpartum: PPARγ^-/F^:MORE^+/Cre^ newborns were not significantly larger at P3 than at P1 (p > 0.06, Fig 1E). In contrast, control littermates gained weight daily between P0 and P3 (p < 0.0003 at each day, Fig 1E) and were 31% larger than PPARγ^-/F^:MORE^+/Cre^ neonates at P3 (p = 0.00001, Fig 1E). Significantly, the difference in birth weight was comparable to the weight of BAT in normal P0 neonates (~100 mg, data not shown) suggesting that other organs attained normal size during embryogenesis in the absence of PPARγ. The majority of these extensively PPARγ deficient neonates either failed to thrive (see Methods for criteria) and were euthanized at P5 or P8 or were less severely affected but found dead at P12 or P13 (Fig 1G, p < 0.0001, log-rank (Mantel-Cox) test compared to non-mutant animals). However, a small number (Fig 1G) survive to weaning thus enabling experiments in adults as previously reported [25, 27].

### Hepatomegaly, hepatitis and hepatocellular necrosis in PPARγ deficient neonates

**Fig 2:**
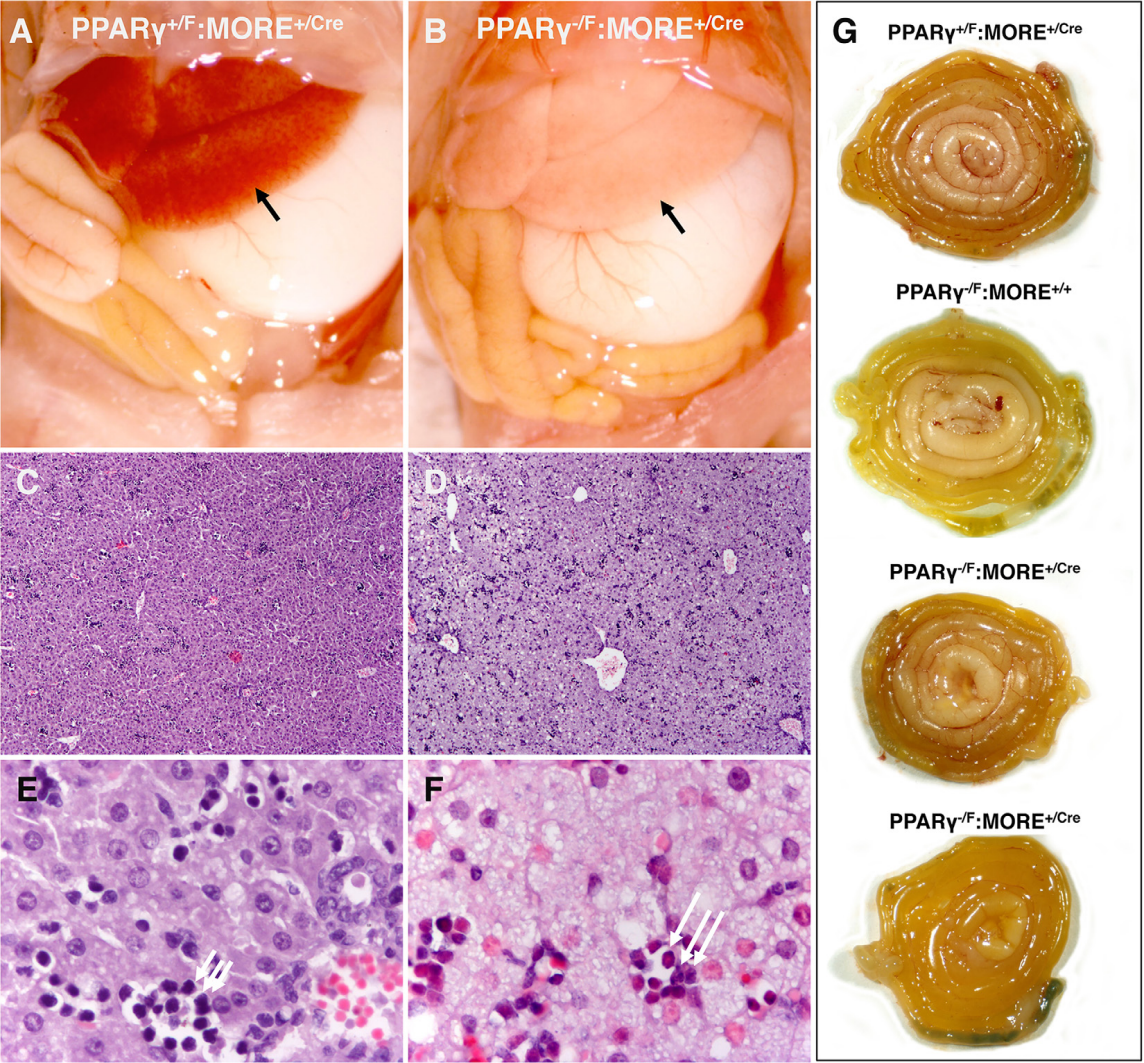
Hepatic steatosis and normal bowel in PPARγ^-/F^:MORE^+/Cre^ neonatal mice. (A and B) The abdominal contents reveal pale, enlarged liver (arrows) in P3 PPARγ^-/F^:MORE^+/Cre^ mice (B) compared to normal controls (A). (C-F). H & E histology of the liver reveals microvessicular steatosis and focal hepatitis in mutant mice (D and F) but absence of steatosis or inflammation and presence of normal extramedullary hematopoiesis in control mice (C and E). Short and long arrows in E and F distinguish normal extramedullary hematopoiesis (E) from acute inflammation (F with neutrophils) in control (E) and PPARγ^-/F^:MORE^+/Cre^ (F) mice, respectively. (G) Gastrointestinal tracts were dissected from duodenum to rectum, loosely coiled on a flat surface with duodenum central and rectum peripheral, and photographed. PPARγ-deficient small and large bowel is normal without evidence of ischemia, hemorrhage, inflammation or necrosis.

Livers of mutant mice were normal size and appearance at birth (not shown). However, P3 mutant mice showed marked hepatomegaly (Fig 1F) with macroscopic pallor (Fig 2A and 2B), microvessicular steatosis, and hepatocellular necrosis with associated acute hepatitis and hemorrhage (Fig 2C–2F and not shown). Extramedullary hematopoiesis, normal at this age (Fig 2C and 2E arrows) was greatly diminished or absent in mutant mice (Fig 2D and 2F). Instead, focal collections of neutrophils were frequent in mutant liver (Fig 2F arrows) immediately adjacent to steatotic hepatocytes reflecting active hepatitis. Pancreata of mutant mice were normal size at P3 (Fig 1F).

Small and large bowel were grossly normal in mutant neonates (Fig 2G) without gross or histologic evidence of ischemia, hemorrhage or necrosis (Fig 2G and not shown). The differences in gross appearance of bowel are consistent with normal variation and variable bowel contents.

### Severe lipodystrophy with lipemia and hyperlipoproteinemia in PPARγ deficient neonates

**Fig 3:**
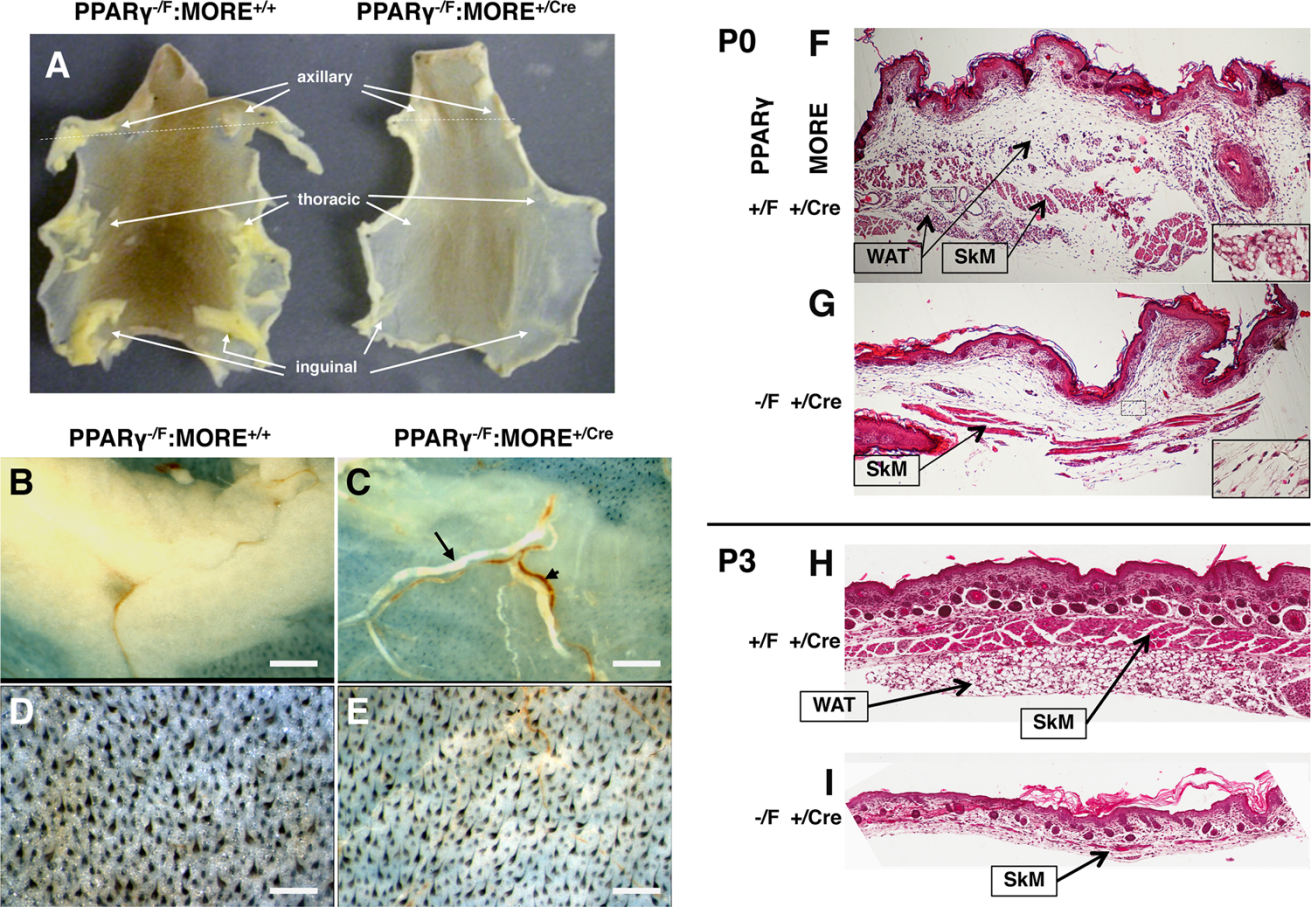
Lipodystrophy in PPARγ^-/F^:MORE^+/Cre^ neonatal mice. Axillary, inguinal and subcutaneous WAT is significantly diminished and lacks mature adipocytes in PPARγ^-/F^:MORE^+/Cre^ mice. (A-E) Skin was removed intact from euthanized P3 mice, restrained in a flattened position, fixed in 10% NBF and photographed from the deep aspect. PPARγ^-/F^:MORE^+/Cre^ mice show greatly diminished axillary, thoracic and inguinal WAT (arrows in A) that contained wispy fibrovascular connective tissue, decreased mature adipocytes, and normal-appearing blood vessels (A, C, E, G, I) in place of well-formed WAT in controls (A, B, D PPARγ^-/F^:MORE^+/+^; F, H PPARγ^+/F^:MORE^+/Cre^;). Control (D) but not mutant (E) skin also showed refractile collections of subcutaneous adipose tissue that were diffusely distributed both superficial and deep (arrows in F and H) to the panniculus carnosus and were largely lacking in mutant skin (G and I). Dashed lines in A indicate the planes of microtome sectioning used for the histologic images presented in F-I. Long and short arrows in C indicate grossly lipemic and non-lipemic blood, respectively. Dashed rectangles in F and G indicate the areas shown at higher power in the insets. Scale bars: B, C 3 mm; D, E 1 mm.

While the interscapular BAT fat pad was entirely absent in most mutant neonates (Fig 1A–1D) necropsy revealed normal size heart, lung, spleen and kidney at P3 (Fig 1F), In contrast, axillary, inguinal and gonadal WAT was well formed in control P3 neonatal mice (Fig 3A, 3B, 3D, 3F and 3H) but was largely absent in PPAR^+/-^:MORE^+/Cre^ animals (Fig 3A, 3C, 3E, 3G and 3I). In place of WAT were small amounts of fragile, wispy tissue with blood vessels rendered especially prominent in relief (Fig 3C) and lacking histologic evidence of maturing adipocytes (Fig 3G).

Prominent dermal hypopigmentation was also observed in mutant P3 neonates (Figs 1A, 3A, 3D and 3E). In addition, nascent subcutaneous adipose tissue, appreciated grossly in control skin as translucent, refractile collections (Fig 3D) and histologically as fibrovascular tissue with collections of recognizable adipocytes (Fig 3F inset), was also absent or severely reduced in PPARγ^-/F^:MORE^+/Cre^ neonates (Fig 3E and 3G inset). Instead, mutant subcutis often contained small amounts of skeletal muscle (panniculus carnosus) directly opposed to dermis without the intervening and/or surrounding adipocyte collections present in controls. By P3 well-formed subcutaneous adipose tissue was present in controls (Fig 3H) but largely absent in mutants (Fig 3I).

**Fig 4:**
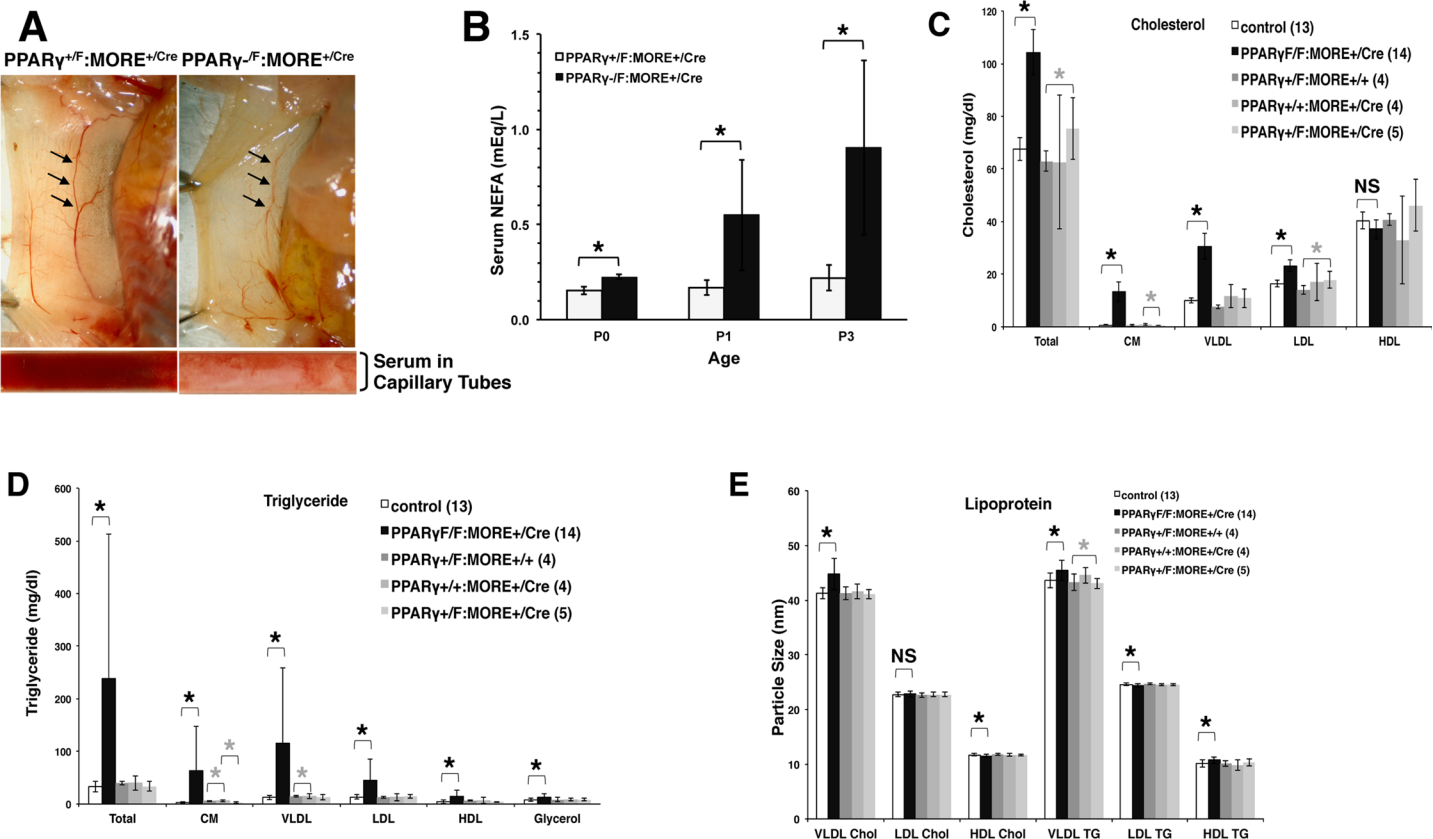
Lipemia with elevated serum lipids and lipoproteins in severely PPARγ deficient PPARγ^-/F^:MORE^+/Cre^ neonatal mice. (A) Grossly lipemic serum. Abdominal skin was reflected and viewed from the deep aspect. Blood in control mice is dark red (left, arrows). Blood in mutant mice is light pink (right, arrows) reflecting lipemia. Gross lipemia was also evident in serum viewed in capillary tubes as prepared from mutant (right) compared to control (left) mice. (B) Serum non-esterified fatty acid levels reveal lipemia in P0, P1 and P3 mutant neonates. (C-E) Serum was analyzed by FPLC followed by quantitative assay for cholesterol, triglyceride, glycerol and lipoprotein particle size. (C.)Total serum cholesterol was elevated approximately 50% in mutant neonatal mice including elevation of cholesterol content in chylomicrons, VLDL and LDL but not in HDL. (D) Total serum triglyceride was elevated almost ten-fold in mutant neonatal mice with significant elevation in triglyceride content of chylomicrons, VLDL, LDL and HDL and elevation of total serum glycerol. (E) VLDL particles carrying cholesterol and triglyceride and HDL carrying triglyceride were 8%, 4% and 6% larger in diameter, respectively, in mutant neonatal mice while other values varied by less than 2%. * p < 0.05; NS, p > 0.05. “Control” indicates combined values for PPARγ^+/F^:MORE^+/+^, PPARγ^+/+^:MORE^+/Cre^ and PPARγ^+/F^:MORE^+/Cre^ neonates. The numbers of mice analyzed of each genotype is indicated in parentheses. Black asterisks, statistically significant difference between PPARγ-deficient neonates and pooled control groups. Grey asterisks, statistically significant difference between the control groups indicated. See also S1 Table.

Mutant neonatal mice were also markedly lipemic. This was immediately apparent as pink/white blood readily observed *in vivo* and *ex vivo* (Fig 3C long arrow, Fig 4A) and was due in part to a 4-5-fold elevation of free fatty acids in serum (Fig 4B and not shown). FPLC and quantitative assay for serum lipoprotein and lipids revealed that total serum cholesterol was also elevated approximately 50% in mutant neonatal mice including elevation of cholesterol content in chylomicrons, VLDL and LDL but not in HDL (Fig 4C). More strikingly, total serum triglyceride was elevated almost ten-fold, with significant elevation in triglyceride content of chylomicrons, VLDL, LDL and HDL and elevation of total serum glycerol (Fig 4D). In addition, lipoprotein particle size was altered in mutants (Fig 4E): VLDL particles carrying cholesterol and triglyceride and HDL carrying triglyceride were 8%, 4% and 6% larger in diameter, respectively, while other values, some showing statistically significant differences, varied by less than 2%. Despite some statically significant differences (Fig 4C–4E, grey asterisks) the three different control genotypes used in these experiments each show the same phenotype relative to the PPARγ-deficient group and are more similar to each other than they are to PPARγ-deficient neonates (Fig 4C and 4E and S1 Table).

### Severe diabetes mellitus with ketosis and relative insulin deficiency in PPARγ deficient neonates

**Fig 5:**
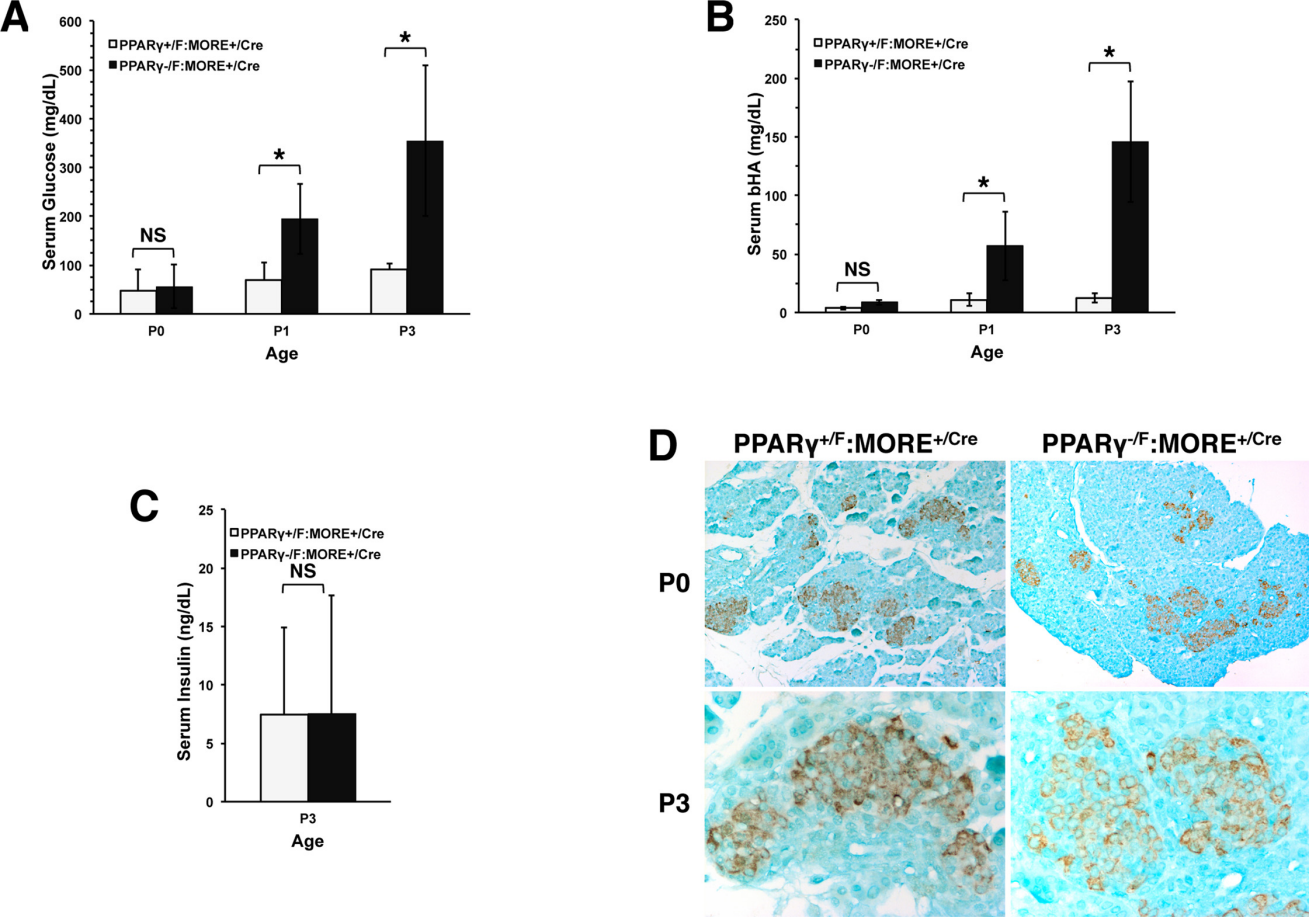
Diabetes mellitus and relative insulin deficiency in severely PPARγ deficient PPARγ^-/F^:MORE^+/Cre^ neonatal mice. (A) Serum glucose levels reveal severe diabetes mellitus in P1 and P3, but not P0, mutant neonates. (B) Serum BHA levels reveal ketonemia in P1 and P3, but not P0, mutant neonates. (C) Normal serum insulin in diabetic P3 mutant mice. (D) IHC for insulin in pancreatic islet beta cells of wild type and severely PPARγ-deficient PPARγ^-/F^:MORE^+/Cre^ neonatal mice. Comparable staining is observed in pancreas from P0 (upper panels) mutant (right) and control (left) mice. At P3 (lower panels), pancreas from mutant mice (right) shows less intense and more diffuse staining for insulin than pancreas from control mice (left). Mutant pancreas reproducibly showed greener counter staining than wild type likely reflecting systemic effects on pancreatic cells in mutant neonates even at P0. In A-C: * p < 0.05; NS, p > 0.05.

PPARγ-deficient neonates were severely diabetic beginning at P1 (serum glucose at P3 355 +/- 155 mg/dL in MORE^+/Cre^:PPARγ^-/F^, n = 7 *vs*. 91 +/- 11 mg/dL in MORE^+/Cre^:PPARγ^+/F^, n = 6, p = 0.0008) with ketonemia (Fig 5A and 5B, respectively). Although insulin resistance was anticipated in these PPARγ-deficient, severely lipodystrophic animals, circulating insulin levels were “inappropriately” normal (7.6 +/- 10.1 ng/ml in MORE^+/Cre^:PPARγ^-/F^, n = 7, vs. 7.4 +/- 7.4 ng/ml in MORE^+/Cre^:PPARγ^+/F^, n = 6. P = 0.98) (Fig 5C). In addition, while pancreatic islets were approximately normal in size and distribution (Fig 5D) IHC revealed decreased intensity insulin staining in presumptive pancreatic islet beta cells of PPARγ^-/F^:MORE^+/Cre^ compared to control neonates at P3 (Fig 5D bottom) while insulin staining was more comparable at P0 (Fig 5D top).

## Discussion

Despite numerous studies of tissue-restricted and isoform-specific functions of PPARγ determining the integrated functional consequences of this pleiotropic regulator of adipogenesis, insulin sensitivity and glucose homeostasis *in vivo* has remained problematic because PPARγ-deficient placental failure uniformly causes embryonic lethality [8–12, 15–23]. To address this important issue we generated mice with global PPARγ deficiency using a targeted Cre “knock-in”/null allele of Mox2 (MORE^cre^) that unexpectedly is expressed in derivatives of epiblast but not trophectoderm or primitive endoderm. Such expression, that does not mirror expression of endogenous Mox2 is probably related to sequences retained in the mutant allele including a phosphoglycerate kinase “promoter” used to drive neo^r^ expression in embryonic stem cells [24]. The MORE^Cre^ allele provided a convenient experimental tool to differentiate extraembryonic from embryonic gene expression and produce live born mice with mutations, such as PPARγ^-/-^, that are normally embryo-lethal due to placental failure. Although MORE^Cre/Cre^ mice die young, MORE^+/Cre^ mice (obtained from Dr. Philippe Soriano) used in the experiments reported here exhibited only mild muscular weakness, as reported [24], but normal breeding performance in our colony.

Our observation of survival to birth of PPARγ^-/F^:MORE^+/Cre^ embryos is consistent with intrinsic placental failure [5, 6], rather than effects in embryos per se, as the cause of embryonic lethality in globally deficient PPARγ gestations: functionally heterozygous PPARγ^-/F^ trophoblast in the context of epiblast-restricted expression of MORE^Cre^ rescues null-lethal placental failure. In addition, the compromised survival observed by gestations with heterozygous PPARγ deficiency (Table 1A and 1B) is consistent with the structural abnormalities reported in PPARγ^**+/-**^ placentas that are similar to, but less severe than, those leading to uniform lethality in PPARγ-/- placentas [5, 6]. Future studies to identify embryos undergoing intrauterine demise with PPARγ^**+/-**^ placentas will validate this prediction and help determine why haploinsufficient embryos are underrepresented.

To our knowledge PPARγ^-/F^:MORE^+/Cre^ mice provide the most severe model of global postnatal PPARγ deficiency studied to date: the vast majority of cells in the tissues examined from mosaic PPARγ^-/F^:MORE^+/Cre^ mice are genotypically PPARγ^-/-^ [25] resulting in severe and global functional PPARγ deficiency. The few PPARγ^-/F^:MORE^+/Cre^ mice that survive to weaning allow studies of severe PPARγ deficiency in adults as previously reported [25–27]. Sox2^Cre^, which potentially provides more efficient recombination in epiblast [31], has been used to study PPARγ in placenta [28] and may produce more complete PPARγ deficiency in embryos. However, the low viability of PPARγ^-/F^:MORE^+/Cre^ neonates (Fig 1G) suggests that more efficient recombination by Sox2^Cre^ may also decrease the hypothesized mosaic rescue and result in uniform neonatal lethality thus precluding studies in adults. In this case future comparisons of MORE^Cre^ and Sox2^Cre^ may elucidate the tissues and cell types in which PPARγ is required for survival beyond the neonatal period.

PPARγ^-/F^:MORE^+/Cre^ neonates were immediately recognizable by gross inspection at birth by virtue of their dermal hypopigmentation and absence of an interscapular brown fat pad. They also displayed diminished subcutaneous adipose tissue examined both grossly and histologically. These observations are consistent with the conclusion from numerous previous studies that PPARγ is required to form mature adipose tissue in adults. Small amounts of residual interscapular tissue and small numbers of differentiated adipocytes that were observed in a few PPARγ^-/F^:MORE^+/Cre^ neonates probably resulted from escape from recombination of a small number of PPARγ^-/F^, functionally heterozygous cells that still express PPARγ allowing them to enter and expand within the adipocyte lineages in these mosaic animals. Other cells present at sites normally undergoing adipogenesis may include stromal cells and adipocyte precursors whose differentiation from mesenchymal progenitors does not require PPARγ [9, 22, 32, 33]. Organogenesis was otherwise largely normal in PPARγ^-/F^:MORE^+/Cre^ mice that were only 9% smaller than control littermates at birth, primarily reflecting absence of the interscapular BAT fat pad. However, such mice failed to gain weight despite nursing successfully and eventually died between P5 and P13 with virtual absence of brown and white adipose tissue, severe hepatic steatosis, lipemia, functional insulin deficiency and diabetes with ketosis. The fact that most of these effects were not observed in mice with either global deficiency of PPARγ2 or deficiency of both PPARγ1 and PPARγ2 specifically in adipocytes or several other cell types [11–23] suggests that they reflect previously unappreciated functions of PPARγ1 in one or more tissues either alone or in combination with PPARγ2.

Gastrointestinal bleeding was observed in the viable PPARγ-/- neonate produced by wild type tetraploid rescue [6]. In contrast, none of five PPARγ^-/F^:MORE^+/Cre^ neonates examined in the current report showed ischemia, hemorrhage or necrosis of small or large bowel suggesting that the original observation was not related solely to PPARγ deficiency. However, we cannot exclude the possibility that small numbers of PPARγ-expressing cells present in PPARγ^-/F^:MORE^+/Cre^ mosaic mice are sufficient to rescue a PPARγ-deficient ischemic bowel phenotype. More subtle effects in small and large bowel that might result from PPARγ deficiency remain to be evaluated.

PPARγ^-/F^:MORE^+/Cre^ neonates were diabetic with normal plasma insulin suggesting that although insulin was expressed by pancreatic β-cells quantitative insulin production, glucose sensing and/or insulin secretion was impaired in the absence of PPARγ. Onset of severe lipemia soon after birth likely reflects the paucity of adipocytes and the resultant inability to store the large amounts of triglyceride and cholesterol newly encountered during the transition from low fat intrauterine nutrition to the high fat diet of nursing. Elevated serum lipids are associated with exacerbation of acute pancreatitis (“lipotoxicity”) and lipoapoptosis is a prominent feature of diabetes [34, 35] suggesting that lipemia may contribute to diabetes in PPARγ^-/F^:MORE^+/Cre^ neonates. Defects in insulin secretion have also been reported secondary to absence of BAT [36] suggesting that diminished BAT in PPARγ^-/F^:MORE^+/Cre^ neonates may also impair glucose metabolism *via* effects on insulin release. However, even if insulin is quantitatively decreased in PPARγ^-/F^:MORE^+/Cre^ pancreas this is unlikely to fully explain the inappropriately normal levels of circulating insulin observed in the presence of extreme hyperglycemia (Fig 5A and 5C). In contrast to neonates, PPARγ^-/F^:MORE^+/Cre^ mice that survive to adulthood show normal serum glucose with greatly elevated plasma insulin [25, 26] suggesting insulin resistance and consistent with predictions from cell culture and pharmacologic studies. The reasons for these differences between neonatal and adult PPARγ^-/F^:MORE^+/Cre^ mice are unclear but may be related to factors that allowed the latter mosaic animals to escape neonatal lethality. In addition, mice with PPARγ-deficient pancreatic β-cells show normal glucose homeostasis despite abnormalities in islet mass [21]. It thus seems likely that insulin resistance and defective insulin delivery to the circulation may combine to produce especially severe glucose intolerance in PPARγ^-/F^:MORE^+/Cre^ neonates and that this combination of defects does not persist in those animals that survive to adulthood. For example, changes in PI3K, FOXO or in insulin and IGF1 receptors can have competing effects on glucose and insulin metabolism that could help explain why surviving PPARγ-deficient adults are euglycemic with elevated plasma insulin [25]. However, investigating these and other molecular hypotheses will first require determining which tissues are responsible for these PPARγ-deficient phenotypes. As a practical matter administering exogenous insulin to PPARγ^-/F^:MORE^+/Cre^ neonates may transiently improve glucose tolerance and prevent neonatal lethality thus extending survival of a greater fraction of PPARγ-deficient animals for experimental analysis as adults.

## Conclusions

PPARγ-deficient neonatal mice were rescued from embryonic lethality by epiblast-restricted recombination and showed marked lipodystrophy with diabetes but inappropriately “normal” levels of serum insulin. This constellation of features suggests that PPARγ1 plays previously unappreciated functions during the neonatal period either alone or in combination with PPARγ2 in lipid metabolism, glucose homeostasis and insulin sensitivity.

## Materials and Methods

### Mice

All animal procedures were performed in accordance with the recommendations in the Guide for the Care and Use of Laboratory Animals of the National Institutes of Health and were approved by the Center for Animal Resources and Comparative Medicine at Harvard Medical School. All efforts were made to minimize suffering. Mice bearing the MORE^cre^ allele in a 129/SvSor genetic background [24] and mice bearing a floxed allele of PPARγ in a mixed 129/Sv:C57BL/6:FVB genetic background [37], obtained from Drs. Philipe Soriano and Dr. Frank Gonzalez, respectively, were maintained in a SPF barrier colony on standard lab chow and water ad libitum. Copulation plugs from virgin female mice mated with male mice were detected between 7 AM and 12 pm and defined day 0.5 (P0.5) of gestation. Newborn mice were genotyped on the day of birth. Health status was monitored daily thereafter until P22 and weights were recorded daily on P0-P3 in 20 neonatal animals. Animal suffering and distress were minimized by euthanasia when humane endpoints were met. Animals were euthanized by decapitation when they failed to grow, showed decreased skin turgor reflecting dehydration and were debilitated with decreased mobility that impaired suckling. This occurred at P5 and P8 in 11 and 3 animals, respectively. Two and one PPARγF/-:MOREcre/+ mutant animals were found dead at P12 and P13, respectively without meeting the criteria for euthanasia on the previous day. Three mutant animals that did not meet the criteria for euthanasia survived throughout the observation period and became eligible for experiments as adults. None of the non-mutant animals met the criteria for euthanasia or died during the observation period. Genotypes reported are those at the time of fertilization as deduced from analysis of neonatal tissue biopsies by allele-specific PCR as described previously [38]. Subsequent MORE-Cre-mediated recombination during embryogenesis produced extensive mosaicism at the time of genotype determination in mice bearing both a MORE^Cre^ and PPARγ^F^ allele. Necropsies were performed essentially as described [39].

### Histology and Immunohistochemistry

Tissues were fixed (10% neutral buffered formalin) for 24 hours by immersion after dissection with or without preliminary vascular infusion with 10% neutral buffered formalin beginning in the left ventricle. Eight-μm paraffin sections were stained with hematoxylin and eosin or were immunostained for insulin using DAB as chromogen and Methyl Green as counterstain as described [40]. Images was obtained either using Nikon digital camera or Aperio ScanScope XT at 40 x lens setting by the Tissue Microarray and Imaging Core Facility of the Dana-Farber/Harvard Cancer Center.

### Serum Chemistry

Neonatal mice were euthanized by decapitation using a fresh blade. A capillary micropipette was used to transfer blood from the decapitation site to a 1.5 ml microcentrifuge tube. After 15 minutes tubes were centrifuged at room temperature and the serum layer was transferred to a fresh tube and stored at –8°C until analyzed. Glucose, β-hydroxybutyrate and NEFA were determined by enzymatic assay with chromogenic substrates (Sigma Chemical GAGO20-1KT, MAK041-1KT and MAK044-1KT, respectively). Insulin was determined by radioimmunoassay (Linco Research/EMD Millipore Sensitive Rat Insulin RIA). FPLC, determination of serum lipoproteins, and assay of triglyceride, glycerol and cholesterol was done by LipoSEARCH Analysis at Skylight Biotech, Inc., Tokyo, Japan.

### Statistical analysis

Values presented are mean +/- standard deviation. Weights and serum chemistry were compared using Student’s t-test. Inheritance was compared by Chi-square test and Binomial test for all genotypes and for pairs of genotypes, respectively. Survival was compared by log-rank Mantel-Cox test. P-value of 0.05 was taken to indicate statistically significant differences between the populations sampled. Statistical analyses were performed using GraphPad Prism version 6 software for Macintosh (GraphPad Software, San Diego, CA, USA).

## Supporting Information

S1 Table (related to Fig 4C–4E) Serum lipoprotein and lipids values obtained by FPLC and quantitative assay for individual and pooled control groups and for PPARγ-deficient neonates. The number of mice analyzed of each genotype is indicated in parentheses. These data are presented in graphical form in Fig 4C–4E. Grey backgrounds and white text indicate statistically significant differences between the groups indicated in the table. Some of these neonates are from matings of PPARγ^+/F^:MORE^+/Cre^ females and PPARγ^-/F^ males. In such neonates inheriting maternal MORE^Cre^ it is not possible to determine if their paternal PPARγ allele is PPARγ^-^ or PPARγ^F^ because near-complete recombination renders the latter undetectable as PPARγ^F^. Data from these mice is combined with neonates with unambiguous inheritance of a PPARγ^F^ allele. If the allele in question is designated “N” the problematic genotypes are PPARγ^+/N^:MORE^+/Cre^ and PPARγ^F/N^:MORE^+/Cre^ and their data is combined with unambiguous PPARγ^+/F^:MORE^+/Cre^ and PPARγ^F/F^:MORE^+/Cre^ neonates, respectively.
(PDF)

## Abbreviations

P0, P1, etc.: postnatal day 0, postnatal day 1, etc
NEFA: non-esterified fatty acid
FPLC: fast protein liquid chromatography
WAT: white adipose tissue
BAT: brown adipose tissue
