# Supplementary material for: Lipodystrophy, Diabetes and Normal Serum Insulin in PPARγ-Deficient Neonatal Mice
Source: PLoS One. 2016 Aug 9;11(8):e0160636. doi: 10.1371/journal.pone.0160636 (PMC4978460; doi:10.1371/journal.pone.0160636)
Supplement: S1 Table — The number of mice analyzed of each genotype is indicated in parentheses. These data are presented in graphical form in Fig 4C–4E. Grey backgrounds and white text indicate statistically significant differences between the groups indicated in the table. Some of these neonates are from matings of PPARγ+/F:MORE+/Cre females and PPARγ-/F males. In such neonates inheriting maternal MORECre it is not possible to determine if their paternal PPARγ allele is PPARγ- or PPARγF because near-complete recombination renders the latter undetectable as PPARγF. Data from these mice is combined with neonates with unambiguous inheritance of a PPARγF allele. If the allele in question is designated “N” the problematic genotypes are PPARγ+/N:MORE+/Cre and PPARγF/N:MORE+/Cre and their data is combined with unambiguous PPARγ+/F:MORE+/Cre and PPARγF/F:MORE+/Cre neonates, respectively. (PDF) [file pone.0160636.s001.pdf]

Control Group	Genotype (# animals)	Parameter	Cholesterol (mg/dL)										Triglyceride [mg/dL]												Particle Size											
			Total		CM		VLDL		LDL		HDL		Total		CM		VLDL		LDL		HDL		Glycerol		VLDL Chol		LDL Chol		HDL Chol		VLDL TG		LDL TG		HDL TG	
1	PPARγ+/F:MORE+/+ (4)	mean	62.99		0.64		7.55		14.09		40.71		27.26		2.38		9.86		11.97		3.06		7.70		41.29		22.64		11.79		43.25		24.73		10.11	
		SD		3.81		0.37		0.73		1.67		2.26		3.59		0.67		1.27		2.01		0.91		3.87		1.19		0.40		0.20		1.54		0.15		0.52
		ratio and P vs group 2	1.01	0.49	0.72	0.16	0.65	0.06	0.83	0.23	1.23	0.20	0.69	0.07	0.44	0.01	0.66	0.04	0.91	0.38	0.48	0.15	0.89	0.36	0.99	0.37	0.99	0.34	1.00	0.38	0.97	0.13	1.01	0.11	1.02	0.34
		ratio and P vs group 3	0.84	0.04	1.07	0.40	0.70	0.06	0.79	0.04	0.88	0.16	0.81	0.13	0.84	0.28	0.75	0.13	0.85	0.17	0.83	0.14	0.93	0.40	1.00	0.39	0.99	0.34	1.01	0.23	1.00	0.40	1.01	0.11	0.97	0.27
2	PPARγ+/+:MORE+/Cre (4)	mean	62.59		0.90		11.65		17.05		33.01		39.76		5.43		14.83		13.12		6.39		8.67		41.59		22.77		11.74		44.55		24.55		9.88	
		SD	25.48		0.29		4.34		7.19		16.69		14.09		1.75		4.54		6.99		5.83		3.16		1.35		0.44		0.25		1.45		0.21		0.99	
		ratio and P vs group 3	0.83	0.17	1.49	0.04	1.08	0.38	0.96	0.42	0.71	0.09	1.18	0.23	1.92	0.02	1.13	0.31	0.94	0.40	1.73	0.17	1.05	0.42	1.01	0.26	1.00	0.49	1.00	0.38	1.03	0.05	1.00	0.48	0.95	0.19
3	PPARγ+/F:MORE+/Cre (5)	mean	75.43		0.60		10.83		17.83		46.17		33.67		2.83		13.14		14.02		3.68		8.27		41.10		22.77		11.69		43.05		24.56		10.39	
		SD		11.78		0.11		3.52		3.23		9.77		9.59		1.34		5.10		3.49		0.68		2.90		0.84		0.47		0.15		0.90		0.21		0.69
pool	pooled controls (13)	mean	67.65		0.70		10.07		16.44		40.44		33.57		3.49		12.65		13.11		4.32		8.22		41.31		22.73		11.73		43.57		24.61		10.14	
		SD		15.91		0.28		3.49		4.46		11.61		10.47		1.82		4.30		4.25		3.32		3.03		1.04		0.41		0.19		1.36		0.20		0.72
na	PPARγF/F:MORE+/Cre (14)	mean	104.35		13.31		30.68		23.24		37.13		238.31		63.13		114.99		45.45		14.74		13.47		44.80		22.81		11.55		45.45		24.32		10.80	
		SD		31.97		14.12		18.25		7.96		13.71		275.02		84.36		143.70		40.18		12.39		6.11		2.87		0.52		0.31		1.84		0.32		0.61
		ratio and p vs pooled controls	1.54	0.02	18.91	0.001	3.05	0.0001	1.41	0.04	0.92	0.06	7.10	0.004	18.10	0.005	9.09	0.005	3.47	0.003	3.41	0.002	1.64	0.002	1.08	0.00004	1.00	0.19	0.98	0.02	1.04	0.00	0.99	0.01	1.06	0.01
		ratio and p vs control group 1	1.66	0.01	20.71	0.049	4.06	0.0123	1.65	0.02	0.91	0.31	8.74	0.076	26.58	0.089	11.67	0.086	3.80	0.061	4.82	0.042	1.75	0.048	1.08	0.01612	1.01	0.28	0.98	0.09	1.05	0.02	0.98	0.01	1.07	0.03
		ratio and p vs control group 2	1.67	0.01	14.87	0.052	2.63	0.0298	1.36	0.09	1.12	0.31	5.99	0.089	11.64	0.100	7.75	0.096	3.46	0.068	2.31	0.108	1.55	0.078	1.08	0.02445	1.00	0.44	0.98	0.15	1.02	0.19	0.99	0.10	1.09	0.02
		ratio and p vs control group 3	1.38	0.03	22.18	0.032	2.83	0.0148	1.30	0.08	0.80	0.10	7.08	0.060	22.31	0.068	8.75	0.069	3.24	0.052	4.00	0.033	1.63	0.044	1.09	0.00631	1.00	0.44	0.99	0.18	1.06	0.01	0.99	0.07	1.04	0.11

S1 Table: Serum lipoprotein and lipids values obtained by FPLC and quantitative assay for individual and pooled control groups and for PPARγ-deficient neonates. The number of mice analyzed of each genotype is indicated in parentheses. Grey backgrounds and white text indicate statistically significant differences between the groups indicated. Some of these neonates are from matings of PPARγ$^{+/F}$:MORE$^{+/Cre}$ females and PPARγ$^{-/F}$ males. In such neonates inheriting maternal MORE^{Cre} it is not possible to determine if their paternal PPARγ allele is PPARγ^{-} or PPARγ^{F} because near-complete recombination renders the latter undetectable as PPARγ^{F}. Data from these mice is combined with neonates with unambiguous inheritance of a PPARγ^{F} allele. If the allele in question is designated “N” then data from PPARγ$^{+/N}$:MORE$^{+/Cre}$ and PPARγ$^{F/N}$:MORE$^{+/Cre}$ neonates is combined with data from unambiguous PPARγ$^{+/F}$:MORE$^{+/Cre}$ and PPARγ$^{F/F}$:MORE$^{+/Cre}$ neonates, respectively.

S1 Table
